# Effectiveness of Several GRAS Salts against Fungal Rot of Fruit after Harvest and Assessment of the Phytotoxicity of Sodium Metabisufite in Treated Fruit

**DOI:** 10.3390/jof10050359

**Published:** 2024-05-17

**Authors:** Mohamed Bechir Allagui, Mouna Ben Amara

**Affiliations:** Laboratory of Plant Protection, National Institute for Agronomic Research of Tunisia (INRAT), University of Carthage, Rue Hedi Karray, Ariana 2080, Tunisia; benamaramouna@gmail.com

**Keywords:** control, decay, GRAS salts, postharvest, sodium metabisulfite

## Abstract

This study evaluates the efficacy of the salts sodium metabisulfite (SMB), ammonium bicarbonate, sodium bicarbonate, and potassium dihydrogen orthophosphate first in vitro against the main postharvest fruit rot fungi, *Alternaria alternata*, *Botrytis cinerea*, *Penicillium italicum*, and *Penicillium digitatum*. Results showed that 0.2% SMB completely inhibited the mycelium growth of the fungal species. Ammonium bicarbonate and sodium bicarbonate were less effective at 0.2% in inhibiting mycelial growth, ranging from 57.6% to 77.6%. The least effective was potassium dihydrogen orthophosphate. Experiments were also performed in vivo on wounded apples inoculated with the most pathogenic fungus, *B. cinerea*, and treated with SMB at concentrations of 0.2, 0.5, 1, 2, and 3%, both preventively and curatively. Results based on the decay size showed that SMB, when used as a preventive treatment, had a reduced efficacy, even with the highest concentration. However, this salt proved to be very effective at 0.5% in curative treatment since the decay was completely blocked. Our results suggest that the appropriate concentration of SMB for post-harvest treatment is 0.5% as a curative treatment. On the other hand, the 1% dose induced the onset of phytotoxicity around the wound. To assess the extent of the phytotoxicity reaction, higher concentrations of 1–4% SMB were applied to wounded fruit. Apples and oranges were inoculated or not with *B. cinerea* and *P. digitatum*, respectively. Doses of 1–4% induced phytotoxicity in the form of a discolored ring surrounding the wound on the epidermis of the fruit; this phytotoxicity enlarged as the concentration of SMB increased. The phytotoxic features were similar on apples and oranges. The methodological procedure made it possible to carry out a quantitative assessment of SMB phytotoxicity. This method is proposed as an easy-to-use technique for quantitatively estimating the phytotoxicity of antifungal compounds on post-harvest fruit.

## 1. Introduction

Fresh fruit and vegetables still contain a high amount of water after being harvested, leading to metabolic processes, such as ripening and senescence. This relatively high water content is likely to play a key role in increasing the susceptibility of fruit to the fungi responsible for decomposition and the often high levels of losses and waste [1]. Injuries on fruit during harvesting, postharvest handling, and commercialization are the primary causes of fungal infections [2]. Postharvest control of fruit rot has relied on synthetic fungicides. However, resistant strains and increased residues in treated fruit are the main factors limiting their use [3,4].

Alternatives are increasingly required to control postharvest diseases including the use of safe low-toxicity compounds, such as natural products with low environmental impact. Organic and inorganic salts, generally recognized as safe (GRAS) ingredients, are considered antifungal products possessing properties as postharvest fruit preservatives [5]. For that, GRAS salts were approved by the United States Food and Drug Administration (USFDA) and by the European Food Safety Authority (EFSA) allowing its use in food conservatives [2,6].

GRAS salts such as carbonates, bicarbonates, sorbates, benzoates, parabens, and metabisulfites, used as aqueous solutions or ingredients of edible coating, are widely cited for controlling postharvest diseases [7,8,9,10,11,12]. Sulfite groups, including sulfur dioxide (SO_2_) (E-numbers E 220–228), are currently approved for some uses on entire fresh fruit. Sulfur dioxide and sulfites are authorized as food additives in the EU [13]. The recent literature suggests that sulfur-containing salts, such as metabisulfites, are effective in controlling important postharvest fruit diseases [14,15,16,17]. For instance, the GRAS salts sodium carbonate and sodium bicarbonate were reported fungistatic compounds able to reduce temporary postharvest decay without detectible phytotoxic effects on treated fruit [18]. These salts applied singly on fruit were effective against green mold on lemons and oranges but their activity was least on infected clementine and mandarins [12,19]. Moreover, the application of hydrogen peroxide (20 g L^−1^) followed by potassium phosphite (20 g L^−1^) controlled both blue and green molds on ‘Eureka’ lemon fruit [20].

The objectives of the study were the (i) evaluation of the in vitro antifungal activity of four GRAS salts against *A. alternata*, *B. cinerea*, *P. digitatum*, and *P. italicum*, (ii) assessment of the in vivo preventive and curative activity of the most promising salt to control decay on apple fruit, and (iii) identification of the signs of phytotoxicity in treated apples and oranges, for which a quantitative assessment was performed.

## 2. Materials and Methods

### 2.1. Fungal Species

**Figure 1:**
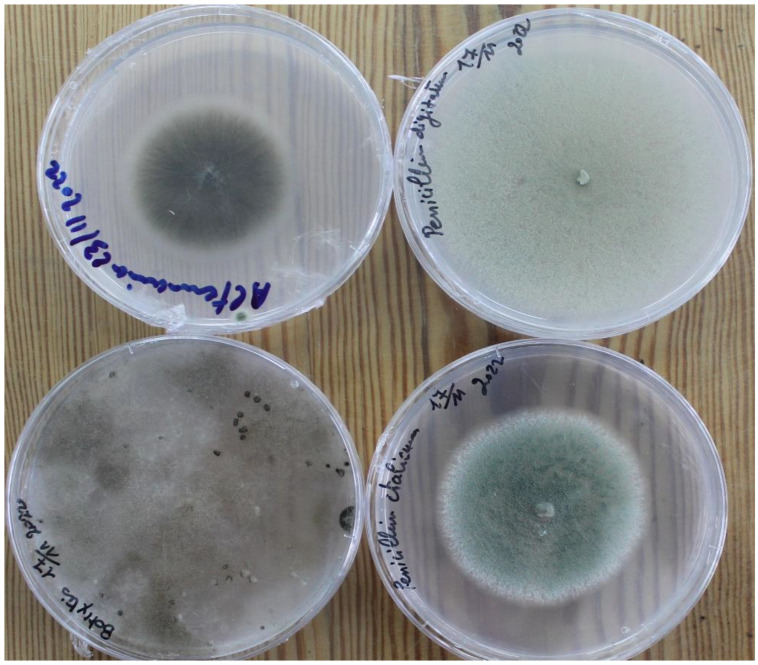
Mycelial growth of the fungal species *Penicillium digitatum* (green mold, **top right**), *Alternaria alternata* (black rot, **top left**), *Penicillium italicum* (blue mold, **bottom right**), and *Botrytis cinerea* (gray mold, **bottom left**) in the artificial medium PDA in which they were maintained for further use [21].

Isolates of *A. alternata*, *B. cinerea*, *P. digitatum*, and *P. italicum* (Figure 1) were obtained from decayed fruit (mandarin and apple) from the local markets ‘Le Kram’ and ‘Grombalia’ (Tunisia). Isolates were identified at the level of species and conserved in PDA medium at room temperature (around 20 °C for 7–14 d in obscurity) for further inoculation.

### 2.2. GRAS Salts Tested In Vitro

Sodium metabisulfite (SMB), ammonium bicarbonate (AMB), sodium bicarbonate (SB), and potassium dihydrogen orthophosphate (PDO) were first tested in vitro (PDA) against the main fungal species of postharvest fruit decay above cited. PDA medium was amended with the respective salt at 0.2% before autoclaving at 120 °C for 20 min. PDA without salt was served as a control. For 7- to 14-day-old cultures of each isolate, a mycelial plug (10 mm diameter) was placed in the center of the medium in 90-mm plastic Petri dishes, then incubated at 20 ± 2 °C in obscurity.

Radial mycelial growth was determined in each Petri dish by measuring two perpendicular diameters of the fungal colony. These measurements were taken after 4, 7, and 9 days of incubation depending on the mycelial growth rate of the fungal species. Three replicates (three Petri dishes) were used for each salt and fungal pathogen. The results were expressed as a percentage inhibition of mycelial growth using the following formula:

Mycelium inhibition (%) = [(dc − dt)/dc] × 100,

where dc = average diameter of the fungal colony on control plates (mm)and dt = average diameter of the fungal colony (mm) on Petri dishes amended with each salt.

### 2.3. GRAS Salt Tested on Fruit

The fruits used in the experiments were apples (*Malus domestica* L.) var. ‘Golden’ and oranges (*Citrus sinesnsis* L.) var. ‘Maltaise’, which were purchased from a supermarket in Ariana (Tunisia). The fruits were selected without wounds, with a healthy appearance, randomized, and their surfaces were disinfected by immersing them for 4 min in 0.72% sodium hypochlorite, rinsed twice with sterilized distilled water, and left to air dry.

**Figure 2:**
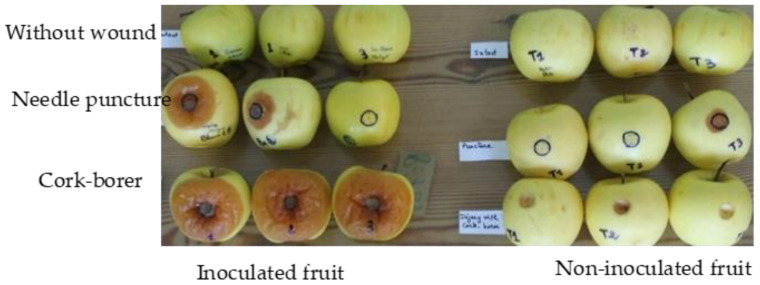
Importance of wound type allowing a better growth of *B. cinerea* on apple fruit.

A preliminary trial was undertaken in order to determine the appropriate kind of wound on fruit that allows rapid fungal progression. The fruit wounds were either 10 mm in diameter using a cork-borer, or five adjacent small punctures (within a 10 mm diameter circle) using a sterilized needle, with the control fruit intact. Figure 2 shows that a 10 mm diameter cork-borer wound was the most appropriate, as the larger the wound, the greater the diameter of the rot and its certainty of development. This type of wound was used in subsequent trials.

Accordingly, fruit was wounded twice in the equatorial diameter in opposite positions using a cork-borer (10 mm diameter, 2 mm depth) immediately before treatment or inoculation. The inoculation consists of a fungal mycelium 10 mm in diameter from the edge of a growing colony, which is placed upside down in each wound. The most effective salt in the in vitro experiment, sodium metabisulfite, was used in the following bioassays at different concentrations. For the curative treatment, apples inoculated with *B. cinerea* were incubated in an ambient room (temperature around 16 °C) for 24 h, then dipped for 1 min in SMB solutions at concentrations of 0.5, 1, 2, or 3% and incubated at room temperature for 7 days. For the preventive treatment, the fruits were first treated with SMB at the concentrations mentioned above and kept at room temperature for 24 h, then inoculated with *B. cinerea* and incubated for 7 days at the same room ambient. In all cases, the fruits were completely soaked for 1 min in a precise dose of SMB solution in recipients of 1 L each. Fruit soaked for 1 min in distilled water served as the positive control and the negative control was fruit soaked in a solution (4 mL/L) of the Syngenta commercial fungicide ‘Celest extra’ co-formulated of fludioxonil (25 g/L) and difenoconazole (25 g/L). The new concentration of the fungicide after this dilution is 0.1 g/L (0.01%) for each of the two active ingredients.

Three replicates (three fruit) per treatment were used and the decay diameter was measured after 7 days of incubation. Fruit rot diameter (mm) was measured twice perpendicularly (to maintain an average measurement per fruit) using a ruler. The percentage of mycelial inhibition was calculated as described below.

% inhibition = [(dc − dt)/dc] × 100,

where dc = average diameter of the control fruit and dt = average diameter of the treated fruit.

### 2.4. GRAS Salt and Phytotoxicity

‘Golden’ apples and ‘Maltaise’ oranges were wounded equatorially on two diametrically opposite sides. A first lot of fruit was inoculated by depositing a plug of mycelium (10 mm in diameter and 2 mm deep) of *B. cinerea* for the apples and of *P. digitatum* for the oranges, placed in the wound of the same size as the plug to completely cover the wound. After two hours, the inoculated fruit was immersed for 1 min in solutions, with each containing 0.5, 1, 2, 3, or 4% SMB. The second lot (non-inoculated) was wounded in the same way and then dipped for 1 min in the SMB solutions mentioned above. The wounded fruits of the control were dipped in distilled water only. The fruits were incubated at an ambient temperature of 16 ± 2 °C. Each treatment contained three fruits, with one fruit being a replicate. The rot diameter on each fruit was measured (in mm using a ruler) twice perpendicularly to determine an average measurement of the rot per fruit.

The phytotoxicity, called a ‘burn or blast of cells’, caused by the application of SMB to the fruit, was assessed at the end of the incubation period when rots reached the apical and/or the peduncle fruit surface. Oranges and apples were incubated for 6 days and 8 days, respectively. The phytotoxicity of SMB on wounded fruit was estimated on the basis of the rot diameter (Figure 6). For that, we first calculated

Decay inhibition (%) = [1 − (D_SMBI_/D_c+_)] × 100

where D_SMBI_ is the decay diameter of wounded, inoculated, and treated fruit. D_c+_ is the diameter of wounded and inoculated but untreated fruit (positive control).

To distinguish between the fungal rot and that induced by the salt, the following approach was pursued. As the fruit rot diameter is the sum of the wound diameter and the rot size itself, it is more accurate to calculate only the rot size for wounded, inoculated, and treated fruit (named: A) and the rot size for wounded and treated fruit only (named: B).

A (mm) = (D_SMBI_ − D_C−_),

where D_SMBI_ is the decay diameter of wounded, inoculated, and treated oranges or apples and D_c−_ is the diameter of only wounded fruit (negative control).

B (mm) = (D_SMB_ − D_C−_),

where D_SMB_ is the decay diameter of wounded and treated fruit.

The size of the rot caused by the fungus is therefore calculated as (A − B).

### 2.5. Statistical Analyses

Data from all experiments were subjected to analysis of variance (ANOVA). The main factors were GRAS salts and fungal species for the in vitro experiment and treatments (preventive or curative) and salt concentrations for the in vivo experiment. For the phytotoxicity test, the main factors were SMB concentrations and inoculated or non-inoculated fruit.

## 3. Results

### 3.1. In Vitro Experiment

**Figure 3:**
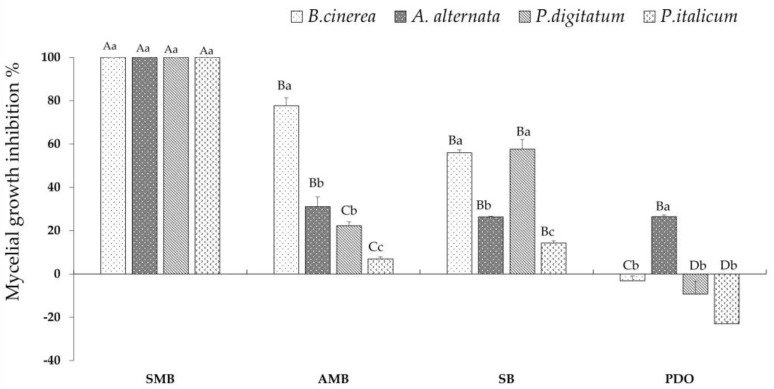
Percentage inhibition of mycelial growth of *B. cinerea*, *A. alternata*, *P. digitatum*, and *P. italicum* on PDA amended with one of the four 0.2% GRAS salts, incubated at 20 °C. Bars with standard error are averages of three replicates. SMB: sodium metabisulfite, AMB: ammonium bicarbonate, SB: sodium bicarbonate PDO: potassium dihydrogen orthophosphate. For *B. cinerea*, mycelial growth was determined after 4 days, for *P. digitatum* after 7 days, and for *A. alternata* and *P. italicum* after 9 days. For each fungus, GRAS salts with different uppercase letters are significantly different and for each GRAS salt, fungi with different lowercase letters are significantly different, according to the Tukey HSD test at *p* ≤ 0.05.

Analysis of variance shows a highly significant effect of GRAS salts, fungi, and the interaction between these two factors on mycelial growth (Table 1). GRAS salts had a strong influence on mycelial growth since the source of variation was high (F = 230.4). Figure 3 shows the inhibition of mycelial growth of *B. cinerea*, *A. alternate*, *P. digitatum*, and *P. italicum* in Petri dishes (PDA) amended with each of the four salts after incubation at 20 °C. SMB at 0.2% (pH = 4.6) completely inhibited the mycelial growth of the four fungal species. For AMB, SB, and PDO, significant differences were observed depending on the target pathogen (Figure 3). AMB (pH = 7.4) differentially inhibited the mycelial growth of *B. cinerea* (77.6%), *A. alternata* (31.1%), *P. digitatum* (22.3%), and *P. italicum* (6.9%). However, SB (pH = 8) was similarly effective in inhibiting mycelial growth in *B. cinerea* (56%) and *P. digitatum* (57.6%), followed by *A. alternata* (26.4%) and *P. italicum* (14.3%). The least effective salt was PDO (pH = 4.9), with a low inhibition of mycelial growth ranging from 26% to −23% (negative value means better growth stimulation than the control).

Furthermore, we carried out an additional test with SMB to check, by lowering this concentration to 0.1% SMB, whether there could be a mycelial growth by any of the fungal species at this lower concentration. It appeared at this concentration of 0.1% that *A. alternata* and *P. italicum* showed limited growth of 20 mm (80 mm control, inhibition rate of 75%), 25 mm (74 mm control, inhibition rate of 66%), respectively, while *B. cinerea and P. digitatum* were totally inhibited at this concentration. SMB therefore proved highly effective at a concentration of 0.2%, irrespective of the fungal species, and was used in subsequent in vivo trials to confirm this efficacy.

### 3.2. In Vivo Activity of Sodium Metabisulfite

**Figure 4:**
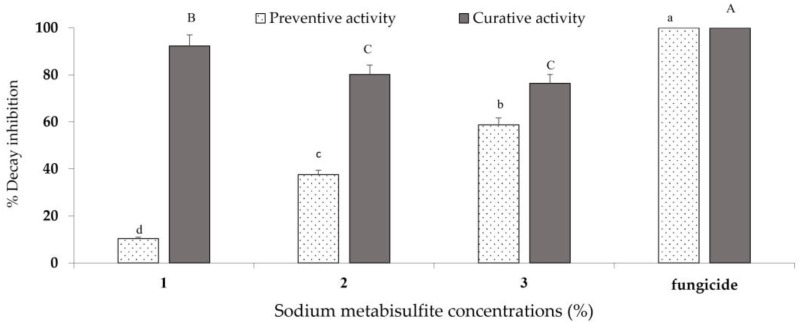
Percentage of rot inhibition by sodium metabisulfite treatment (1–3%) of ‘Golden’ apples inoculated with *Botrytis cinerea*. Fruits were dipped preventively or curatively in different concentrations of sodium metabisulfite and then incubated at room temperature for 7 days. The bars represent the standard error on the mean of three replicates. Upper case letters lower case Means with different letters are significantly different at *p* ≤ 0.05. ANOVA summary: F ratios: treatment (152.9 **), SMB concentration (5.6 *), and interaction treatment × SMB concentration (22.4 **). ** Highly significant at *p* ≤ 0.01, * Significant at *p* ≤ 0.05.

SMB concentrations ranging from 0.2 to 3% were evaluated in vivo as preventive and/or curative treatments. Significant differences between treatments (preventive and curative) and between SMB concentrations were recorded, as indicated in the ANOVA (Figure 4, Legend). The preventive activity of SMB was less effective against *B. cinerea* on apples than the curative activity, due to a low reduction in rot compared with the curative treatment. Thus, the percentage of rot inhibition on apples inoculated and soaked preventively in 2% and 3% SMB was 37.6% and 58.8%, respectively. However, the reduction in rot on apples inoculated and soaked curatively in 2% and 3% SMB solutions was 80% and 76.4%, respectively, compared with the commercial fungicide, which was completely effective (100%) when used preventively or curatively.

**Figure 5:**
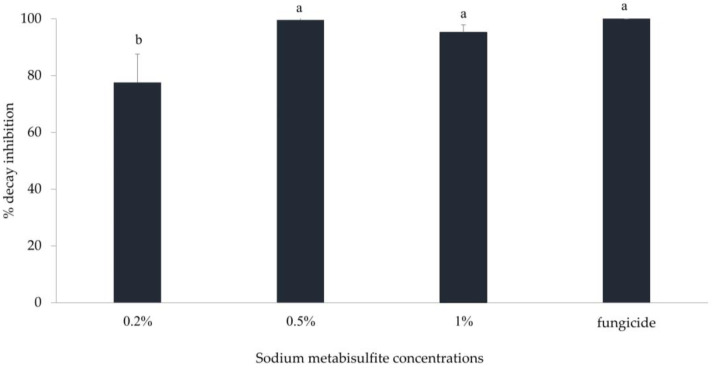
Percentage of rot inhibition in apple fruit var. ‘Golden’ inoculated with *B. cinerea* and curatively dipped in different concentrations (0.2–1%) of sodium metabisulfite then incubated at room temperature for 7 d. Bars represent the standard error on the mean of three replicates. Means with different letters are significantly different at *p* ≤ 0.05. ANOVA summary: F-ratio of SMB concentrations (14.23 *). * Significant at *p* ≤ 0.05.

Increasing SMB concentrations from 1% to 3% had no significant added effect on rot reduction, particularly in the curative treatment. However, a zone of necrosis similar to phytotoxicity was observed around the wound on the fruit skin, which increased with increasing SMB concentrations. As a result, other in vivo trials were conducted with this salt at low concentrations (0.2%, 0.5%, and 1%). When applied at the 0.2% concentration, SMB reduced the size of rot on apples by up to 77.6% (Figure 5). However, the 0.5% concentration completely inhibited rot on inoculated apples (99.6%). Soaking the inoculated apples in a solution containing 1% SMB reduced decay by 95.2% with concomitant mild phytotoxicity on the fruit pericarp, such as whitening and collapse at the inoculation point. Thus, curative treatment with SMB at 0.5% or 1% was the most effective concentration, almost similar to the classical fungicide ‘Celest Extra’ applied at 0.01% for fludioxonil and 0.01% for difenoconazole. SMB was effective against rot caused by *B. cinerea* on apples at the non-apparent-phytotoxic dose of 0.5%.

### 3.3. Phytotoxicity of Sodium Metabisulfite

**Figure 6:**
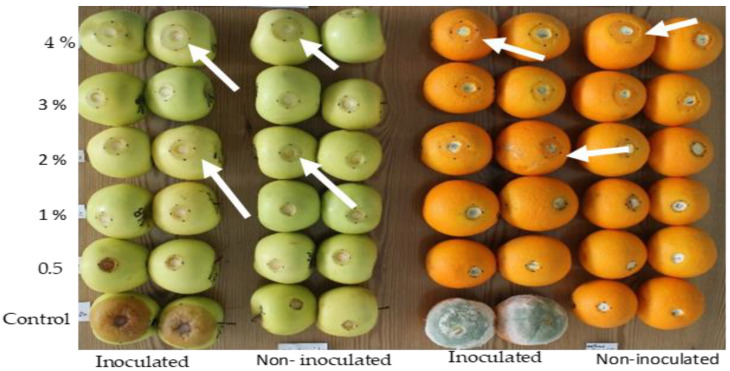
Phytotoxicity (as rot size) of sodium metabisulfite in relation to increasing concentrations from 0.5 to 4% on apples inoculated/un-inoculated with *B. cinerea* (**left**) and on oranges inoculated/un-inoculated with *P. digitatum* (**right**). ANOVA summary of the phytotoxicity in apple fruit: factor SMB concentration: F value = (11.6 *), factor pathogen: F value = (2.6 ns), and interaction SMB concentration × pathogen: F value = (2.6) *. ANOVA summary of the phytotoxicity in orange fruit: factor SMB concentration: F value = (22.4 *), factor pathogen: F value = (0.2 ns), and interaction SMB concentration × pathogen: F value = (0.6 ns). * Significance at *p* ≤ 0.05, ns not significant.

Over time, fruit soaked in SMB solutions showed a translucent circular white rot (Figure 6) that extended in a circular pattern surrounding the wound that had been perforated by the cork-borer. According to the ANOVA in Figure 6 (legend), the concentration of SMB was a significant factor in the induction of this rot zone, while the effect of the pathogen on this rot was not significant.

The size of the rot was measured on oranges and apples inoculated and treated with increasing concentrations of SMB from 0.5 to 4% (Table 2). In this case, rot size ranged from 2.5 to 9.5 mm for apples (8 dpi) and from 0.9 to 14.9 mm for oranges (6 dpi). When the fruits were treated only, rot size ranged from 2.8 to 11.7 mm for apples and from 1.3 to 9.2 mm for oranges. As a result, the percentage of rot inhibition compared to the control (inoculated untreated fruit) decreased with increasing SMB concentration, ranging from 98.7 (0.5% SMB) to 78.2 (4% SMB) for oranges and from 93.2 (0.5% SMB) to 71.2% (4% SMB) for apples.

**Figure 7:**
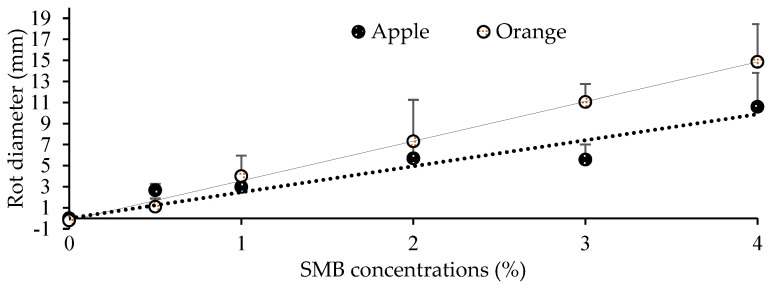
Linear regression between sodium metabisulfite concentration (x-axis) and rot diameter size (y-axis) was measured on oranges (6 dpi) and apples (8 dpi). Each point is the mean diameter of eight replicates (fruit). Vertical bars represent standard deviations.

These results indicate that SMB concentrations appear to be fully effective from 0.5% and that the necrosis produced on fruit treated with concentrations exceeding 0.5% is not due to fungal infection but rather linked to SMB phytotoxicity, which is also confirmed by the ANOVA. To exploit this point, we took, for each concentration, the values for inoculated-treated and non-inoculation-treated fruit as replicates of SMB treatment only in order to determine the average size of the necrosis for each concentration. Such adjustment of the data made it possible to determine a linear regression linking the concentration of SMB (x) to the corresponding average size of the necrosis (y). This gives the equation y = 3.76.x − 0.2 with an R^2^ = 0.99 for oranges and y = 2.47.x with an R^2^ = 0.96 for apples. Figure 7 shows these two straight lines, which fit the measured values perfectly.

**Figure 8:**
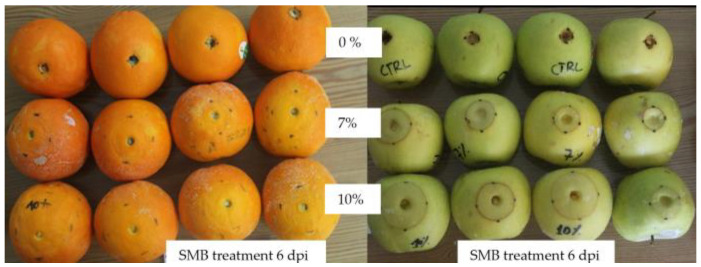
Evolution of the phytotoxic reaction to SMB in the form of circular necrosis around the wound on ‘Golden’ apples and ‘Thompson’ citrus fruit treated with 7% and 10% SMB and incubated at 18 °C for 6 days.

To validate the accuracy of this linear relationship between SMB concentration and necrosis diameter, we performed another trial in which only apples and oranges were wounded and treated with higher concentrations of SMB (7% and 10%), in addition to the control (Figure 8).

The diameter of the necrosis on the fruit for each treatment after 6 days for oranges and 8 days for apples is shown in Table 3, illustrating the values expected according to the two linear formulae mentioned above and those measured. It appears from the Chi-square test that the hypothesis of equality between the observed and expected values is well-validated (null hypothesis accepted).

## 4. Discussion

Our results from in vitro trials using GRAS salts showed that 0.2% AMB induced 77.6% inhibition against *B. cinerea* but showed limited activity against other fungal species. SB was less effective than AMB, with inhibition of 56 and 57.6% against *B. cinerea* and *P. digitatum*, respectively. The PDO was not only ineffective but also induced mycelial growth that was greater than that of the control, particularly in the case of *P. digitatum* and *P. italicum*. Previous results indicated that at a concentration of 0.16–0.50%, ammonium, potassium, and sodium bicarbonates inhibited approximately 95% of the in vitro growth of *B. cinerea* colonies [22]. This inhibition is explained by the buffering capacity of the carbonate, which alkalinizes the environment of pathogens such as *B. cinerea*, which needs an acidic medium to grow properly [23]. Zhao et al. [24] evaluated the antifungal activity of 17 GRAS salts in vitro against 4 postharvest fungal diseases of citrus fruit. Their results, based on inhibition rates, pointed to differential reactions of fungi to the salts, in agreement with our results. Thus, sodium silicate (1%) was 100% inhibitory against all tested fungi. In contrast, sodium carbonate (1%) was 100% effective against *P. digitatum* and *P. italicum* but had no activity against *G. citri-aurantii* or *G. gloeosporioides*. In this respect, Lyousfi et al. [25] evaluated some organic and inorganic salts (food additives) in vitro as antifungal agents against *Monilinia fructigena*. Most additives showed significant inhibition of mycelial growth but the extent of the inhibition varied according to the additive and its concentration (sodium bicarbonate, sodium carbonate, and copper sulfate being the most effective, while ammonium carbonate and citric acid were the least effective).

Sulfur-containing food additives act effectively in vitro against various postharvest fungal diseases. This was observed in our in vitro tests with SMB at 0.2%, which was the most effective, irrespective of the fungal species, as mycelial growth was completely inhibited at this concentration. Kim et al. [26] highlighted that sodium metabisulfite was one of the most effective salts for controlling microbial growth. Tian et al. [27] demonstrated the strong in vitro antifungal activity of potassium metabisulfite and sodium hyposulfite (0.1%) against four kiwifruit soft rot fungi, inhibiting mycelial growth in a concentration-dependent manner. SMB completely inhibited the mycelial growth of *A. alternata* and *B. cinerea* when used at a concentration of 0.2 M (3.8%) [6]. The sulfur salt completely inhibited the mycelial growth of *P. italicum* at 0.4% [9] and of *P. digitatum* at 2% [28]. Inhibition of *P. digitatum* and *P. italicum* by potassium and sodium metabisulfite in a Petri dish at concentrations of 0.19–1.9% has also been reported [5]. Sodium metabisulfite at a concentration of 0.19% completely inhibited mycelial growth of *A. solani* [6]. In another study, 0.01% SMB caused total inhibition of conidial germination and germ-tube elongation in *Venturia inaequalis* [29]; such a concentration, according to our results on mycelial growth, seems to be a very low dose to ensure total inhibition in vitro. Our in vitro results showed that 0.2% SMB is effective in ensuring total inhibition of fungi in vitro but that 0.1% is not sufficient to achieve the same result and is therefore considered a limiting concentration for in vitro efficacy.

With regard to the pH of the 0.2% GRAS salt solutions, SMB and PDO were acidic (pH between 4.6 and 4.9), while AMB and SB were basic (pH between 7.4 and 8). Despite being close in pH, SMB and PDO showed diametrically opposed efficacy, while AMB and SB showed roughly similar efficacy for the mycelial growth of fungal pathogens having different pH preferences. This suggests that the pH of these solutions cannot be considered an essential factor in the inhibition of mycelial growth by these GRAS salts when tested in vitro with various fungal species.

According to Talibi et al. [30], the ability of GRAS salts to control postharvest diseases can be estimated by in vitro experiments but in vivo bioassays are still needed to confirm the in vitro results. In the first part of our in vivo study, inoculated apples were dipped in different concentrations of SMB (1%, 2%, and 3%) as preventive and curative treatments. Results showed that SMB was ineffective when used preventively at all concentrations. However, the 1% curative dip showed high efficacy (92.4%) compared with the commercial fungicide (100%). In the second part of the study, we tested SMB curatively on apples (fruit treatment after fungal inoculation) at concentrations of 0.2%, 0.5%, and 1%. The results showed that SMB, when used curatively at 0.5%, was effective in controlling rot on apples inoculated with *B. cinerea*.

Sulphite salts dissolved in aqueous solutions are released as sulfur dioxide (SO_2_) (67.4%) [13,30] which, in contact with the fungal mycelia, alters the permeability of its membrane [31] and accumulates in the cytoplasm. This disorder inhibits mycelial growth through intercellular reactions [13,32]. Furthermore, it was suggested that contact between the salt solution and the fruit pericarp could modify the pH of the wound in which the fungus develops [5]. Accordingly, the pathogenicity of some fungal pathogens could increase. However, other fungal species using their own mechanisms, such as *Alternaria*, are able to alkalinize the medium in the pericarp infection wound, while others, such as *Penicillium*, can acidify the ambient setting [33,34,35]. With respect to our pH analyses, the SMB solution was acidic, as were the orange and apple juices (pH = 3 and 4, respectively). These environments should be favorable to *Botrytis* and *Penicillium*, which prefer acidic environments. It appears that the SMB, even though acidic, blocked the fungi inoculated on these fruits in a fungistatic manner. This suggests that pH is less important when the treatment product is formulated with an active ingredient with strong antifungal properties.

The ineffectiveness of SMB, when applied as a preventive measure (fruit treatment before fungal inoculation), could be attributed to the gradual dissipation of the sulfur component, so that the subsequent fungal infection escapes its full activity. However, there are reports on other GRAS salts without a sulfur component, such as potassium sorbate, showing that they are also ineffective as a preventative measure. For example, Olmedo et al. [36] reported that potassium sorbate was tested against green and blue mold on lemon fruit inoculated and treated preventively and curatively. They pointed out that no efficacy was observed as a preventive measure, whereas curative treatment was effective. The same observation was underlined when thermal treatments were used. In fact, Lydakis and Aked [37] reported that ‘Sultanina’ table grape berries inoculated with *B. cinerea* after vapor heat treatment (preventive treatment) were more susceptible to the fungal disease than the control. Nevertheless, curative heat treatments applied after inoculation significantly reduced infection compared to the control. Spadoni et al. [38] found that the treatment of peach fruit with hot water as a preventive measure failed to control brown rot caused by *Monilinia fructicola*, unlike curative treatment, which demonstrated high curative efficacy against *M. laxa* [39]. The ineffectiveness of the preventive treatment was explained by an increased emission of volatile organic compounds, such as acetaldehyde and ethanol, from the fruit after a short period of the treatment. These volatile compounds were linked to a stimulation of conidial germination and an increase in the incidence of brown rot [38]. We can hypothesize that the mechanism described above may be similar to that triggered in fruit treated preventively with SMB in our trials. In a review, Palou et al. [40] considered that the lack of preventive activity is one of the main handicaps of GRAS salts. Our results on the ineffectiveness of preventive treatment of fruit with SMB are in accordance with the previous findings using other means of treatment.

Phytotoxicity of sodium metabisulfite was assessed at concentrations from 0.5 to 4% on apples and oranges. Necrosis was developed on fruit peel around the point of injury even though it was on un-inoculated fruit. This confirms that necrosis is not only produced by pathogenic fungi but also by SMB at concentrations above 0.5%. An increase in the necrosis area was registered even in healthy fruit (fruit wounded and treated but not inoculated). In the literature, salts inducing phytotoxicity on fruit pericarp were described. For example, the application of copper sulfate has shown phytotoxic effects on fruit rinds, leading to visible darkening and sinking at the inoculation point [24]. Martínez-Blay et al. [5] pointed out the phytotoxicity of sodium and potassium metabisulfite as well as aluminum and potassium sulfate on citrus fruit.

We can assume that dipping healthy fruit in high concentrations of SMB would affect the cellular cohesion of the fruit pericarp, which would then allow the fruit to be infected more rapidly. In addition, since the natural antagonists of the microbiome, which are normally latent on the pericarp, would be disrupted after treatment of the fruit, the pathogen would be able to multiply again without mechanical/antagonistic barriers. For this reason, using a higher level of sodium metabisulfite will not improve the inhibition of rot size but will increase the sensitivity of the fruit skin to the side effect of this salt. Our analyses highlighted that the appropriate concentration was 0.5% in the curative treatment without showing any noticeable phytotoxicity on the fruit. Thus, Martinez-Blay et al. [5] reported that the application of SMB at a concentration of 100 mM was phytotoxic on ‘Valencia’ oranges but that 50 mM caused no apparent damage to the rind of the treated fruit and was very effective against mold. The 50 mM concentration (0.85%) is very close to 0.50%, showing efficacy in our trials with no apparent phytotoxic reaction in treated fruit.

To our knowledge, the evaluation of salt phytotoxicity was mainly based on the qualitative visual description of the symptoms appearing after treatment of the fruit. For example, in dip treatments of stone fruit, to control fungal pathogens after harvest using food additives, phytotoxicity on the fruit skin caused by the salts was assessed visually on a scale of 1 to 4, depending on the surface area covered by fungal lesions [41]. Delisle-Houde et al. [42] used a visual scale of 0–4 to assess the severity of phytotoxicity of different salts on the foliar surface of lettuce infected with the bacteria ‘*Pseudomonas cichorii*’, causing a lettuce varnish spot. Our analyses on the severity of phytotoxicity used a quantitative procedure by relating the size of necrosis measured on treated fruit to the corresponding concentration of salt. We suggest using the protocol/methodology applied here as a model for a quantitative analysis of the phytotoxicity of compounds used to treat fruit. This suggestion may become valid after other tests including different compounds on various fruits (apples are the best candidates thanks to the obvious necrosis margin).

## Figures and Tables

**Table 1:** Analysis of variance in the effect of GRAS salts on mycelial inhibition of various fungi.

Source of Variation	DF	F-Value
Factor A (GRAS salts)	3	230.4 **
Factor B (fungi)	3	18.2 *
Interaction A × B	9	10.6 *

** Highly significant at *p* ≤ 0.01, * Significant at *p* ≤ 0.05. GRAS salts were sodium metabisulfite, ammonium bicarbonate, sodium bicarbonate, and potassium dihydrogen orthophosphate. The pathogens were *A. alternata*, *B. cinerea*, *P. digitatum*, and *P. italicum.* DF: degree of freedom.

**Table 2:** Evaluation of the phytotoxicity of SMB by the size of rots on oranges and apples treated with increasing SMB concentrations.

SMB Concentrations % (x)	0.5	1	2	3	4
Oranges (cv. ‘Maltaise’)					
Decay inhibition (%) ^1^	98.7	94.1	88.0	84.8	78.2
A (mm)= D_SMBI_ − D_C−_	0.9 ± 0. 1	4 ± 1.4	8.2 ± 5.6	10.4 ± 1.1	14.9 ± 3.5
B (mm) = D_SMB_ − D_C−_	1.3 ± 0.3	4 ± 2.1	6.4 ± 2.3	11.7 ± 1.5	9.2 ± 2.9
Fungal effect (A − B) (mm)	−0.4	0	1.8	−1.3	5.7
(A + B)/2, [y observed] (mm)	1.1	4	7.3	11.05	12.05
y = a.x +b, [y calculated]	y = 3.76.x − 0.2; R^2^ = 0.99				
Apples (cv. ‘Golden’)					
Decay inhibition (%) ^1^	93.2	91.6	84.7	87.1	71.7
A (mm)= D_SMBI_ − D_C−_	2.5 ± 0.3	2.5 ± 0.6	5.1 ± 1.6	5.8 ± 1.3	9.5± 2.3
B (mm) = D_SMB_ − D_C−_	2.8 ± 0.9	3.5 ± 1.7	6.3 ± 1.4	5.3 ± 1.6	11.7 ± 4.2
Fungal effect (A − B) (mm)	0.3	1.0	1.3	−0.5	2.2
(A + B)/2, [y observed] (mm)	2.7	3.0	5.7	5.6	10.6
y = a.x + b, [y calculated]	y = 2.47.x; R^2^ = 0.96				

^1^ Decay inhibition (%) = [1 − (D_SMBI_/D_c+_)] × 100; D_SMBI_: Decay diameter of inoculated treated oranges or apples; D_SMB_: Decay diameter of un-inoculated treated fruit; D_c+_: Positive control (diameter of untreated, inoculated wounded fruit diameter); D_c−_: Negative control (diameter of untreated, un-inoculated wounded fruit).

**Table 3:** Mean values of rot diameter (mm) measured and those expected from the respective linear regressions of apples and oranges in relation to SMB concentrations and Chi-square test comparing the critical and calculated Chi-square values.

	SMB (%)	Observed Values	Expected Values	Chi-Square Test
Oranges cv. ‘Thompson’	7	26.02	26.12	χ^2^_c = 0.809_ χ^2^_(0.05, 1) = 3.84_
	10	31.9	37.4	
Apples cv. ‘Golden’	7	16.7	17.3	χ^2^_c = 0.23_ χ^2^_(0.05, 1) = 3.84_
	10	22.4	24.7	

## Data Availability

Data are contained within the article.
